# Work–Family Balance and Presenteeism‐Related Productivity Loss in Emergency Nurses: Cross‐Sectional Indirect Associations Through Burnout Dimensions and Fatigue

**DOI:** 10.1155/jonm/7344295

**Published:** 2026-10-26

**Authors:** Fangyun Luan, Jingzheng Yan, Wenlong Sun, Xiao Han, Yingjuan Cao

**Affiliations:** ^1^ Department of Emergency Medicine Qilu Hospital of Shandong University Jinan China qiluhospital.com; ^2^ Department of Nursing Qilu Hospital of Shandong University Jinan China qiluhospital.com; ^3^ Nursing Theory & Practice Innovation Research Center Shandong University Jinan China sdu.edu.cn; ^4^ School of Nursing & Rehabilitation Shandong University Jinan China sdu.edu.cn

**Keywords:** cross-sectional study, depersonalization, emergency nurses, emotional exhaustion, fatigue, personal accomplishment, presenteeism, work–family balance

## Abstract

**Aim:**

To estimate associations of work–family balance with presenteeism‐related productivity loss and to examine cross‐sectional statistical indirect associations through the three MBI‐HSS burnout dimensions and fatigue among emergency nurses.

**Background:**

Emergency nursing combines time‐critical care, rotating schedules, and emotional demands. The MBI‐HSS defines emotional exhaustion, depersonalization, and personal accomplishment as distinct dimensions; a single composite can conceal different associations.

**Methods:**

We conducted a cross‐sectional secondary analysis of deidentified baseline data from 3597 emergency nurses in the Chinese Nurses’ Health Cohort (TARGET). The three MBI‐HSS dimensions were scored separately and entered simultaneously. Pearson correlations, hierarchical linear regression with HC3 robust standard errors, a joint Wald test, and a generalized indirect‐association model with 5000 bootstrap resamples were used.

**Results:**

Work–family balance correlated inversely with productivity loss (*r* = −0.399). The three burnout dimensions were jointly associated with productivity loss (HC3 Wald *F* = 99.77, *p* < 0.001). The adjusted total association was *B* = −0.307 (95% bootstrap CI −0.328 to −0.285), and the association conditional on the three dimensions and fatigue was *B* = −0.163 (95% bootstrap CI −0.191 to −0.137). The total statistical indirect association was −0.1435 (95% bootstrap CI ‐0.1621 to −0.1257), representing 46.8% of the total association. The combined dimension‐only component was −0.0890 (29.0%), the fatigue‐only component was −0.0338 (11.0%), and the combined serial component through a burnout dimension followed by fatigue was −0.0206 (6.7%).

**Conclusion:**

Better work–family balance was associated with less presenteeism‐related productivity loss. Emotional exhaustion accounted for the largest dimension‐specific indirect component; depersonalization was smaller, and the personal‐accomplishment component operated in the opposite direction. These cross‐sectional decompositions do not establish temporal mediation or causation.

**Implications for Nursing Management:**

The findings support prospective evaluation of scheduling predictability, recovery time, staffing adequacy, and family‐supportive supervision as organizational strategies. They do not establish intervention effectiveness.

## 1. Introduction

Emergency departments expose nurses to fluctuating patient volume, time‐critical decisions, rotating schedules, and repeated emotionally demanding encounters. These conditions can compete with family responsibilities and recovery outside work. Work–family balance is broader than low conflict: it reflects whether demands in one domain interfere with the other and whether resources gained in work or family facilitate functioning across domains. The 14‐item measure used here, therefore, includes work interference with family, family interference with work, work‐to‐family facilitation, and family‐to‐work facilitation [1–8].

Conservation of resources (COR) theory and the Job Demands–Resources (JD–R) model provide complementary, rather than interchangeable, explanations. COR theory proposes that people seek to retain and replenish valued resources; recurrent conflict can initiate resource‐loss cycles, whereas family support, control, and recovery opportunities can protect resources [9, 10]. The JD–R model locates those processes within working conditions: high demands consume capacity, while job and personal resources can buffer strain and support engagement [11, 12]. In emergency nursing, work–family balance may, therefore, indicate access to recovery and support while also reflecting how demands are organized.

Burnout is an occupational response described by three related but distinct MBI‐HSS dimensions: emotional exhaustion, depersonalization, and personal accomplishment [13–15]. Nursing studies link work–family conflict with burnout [16–20], and emergency‐nursing research shows substantial variation across burnout‐related outcomes [21–30]. Because a single composite can obscure dimension‐specific patterns, we analyzed the three dimensions separately. Fatigue is related but distinct; it includes physical and mental depletion and is associated with psychosocial strain, irregular shifts, overtime, musculoskeletal symptoms, and patient‐safety risk [31–38].

Presenteeism‐related productivity loss describes impaired performance while a person remains at work. It is less visible than absence and may involve reduced concentration, energy, task completion, or efficiency [39–47]. Burnout dimensions and fatigue may show different associations with work functioning, so treating them as interchangeable measures would weaken interpretation.

We, therefore, examined whether work–family balance was associated with presenteeism‐related productivity loss and decomposed that association through emotional exhaustion, depersonalization, personal accomplishment, and fatigue. Regression‐based multiple‐mediator methods can separate nonoverlapping statistical components [48, 49], and previous research has linked occupational strain with presenteeism‐related outcomes [50]. We expected better work–family balance to be associated with lower emotional exhaustion and depersonalization, greater personal accomplishment, less fatigue, and lower productivity loss. Because all variables were measured at the same survey occasion, the analysis estimates associational decompositions and does not test temporal mediation or causation.

## 2. Methods

### 2.1. Study Design, Setting, and Participants

We used baseline data from the Chinese Nurses’ Health Cohort (TARGET), which was developed by expanding the sample size and geographical recruitment scope of the Shandong Nurses’ Health Cohort. Data used in the present study were collected between February 2021 and October 2025. Participant recruitment and data collection began after the provincial ethics approval document (No. KYLL‐202011‐085) was issued on 1 February 2021. The cohort master contained 64,114 nurses from 16 provinces across China. Details of TARGET have been reported previously [51]. Recruitment combined online and in‐person project briefings. The study team entered cooperation agreements with participating hospitals, and participants provided electronic informed consent. Local coordinators then distributed electronic questionnaires through the China Nurses’ Health Cohort WeChat platform developed by the study team. Data were stored on an independent cloud server and MySQL database cluster. Authenticated data managers could access the TARGET data platform for interface calls, maintenance, and data‐status monitoring; direct access was restricted to trained personnel who had signed the relevant data‐use agreements [51].

Registered nurses aged 18–65 years who were employed at a participating hospital were eligible. Nurses were ineligible if they were retired, undertaking training or further study, had worked for less than 6 months, reported a severe physical or mental disorder, or were on leave during data collection. The final analytic sample comprised 3597 emergency nurses.

### 2.2. Measures

#### 2.2.1. Work–Family Balance

Work–family balance was measured with the 14‐item Work–Family Balance Scale, developed from work–family interface research by Grzywacz and Marks and adapted for Chinese respondents by Zeng and Yan [2, 5]. Items are rated from 1 to 5. Work‐interference‐with‐family items 1–4 and family‐interference‐with‐work items 8–11 were reverse scored as 6 minus the response; the remaining facilitation items retained their original direction. Total scores range from 14 to 70, with higher scores indicating a more favorable balance of low interference and greater facilitation. Cronbach’s alpha was 0.745 in this sample.

#### 2.2.2. Job Burnout

Job burnout was assessed with the Chinese 22‐item Maslach Burnout Inventory‐Human Services Survey [13–15, 52]. Responses range from 0 (*never*) to 6 (*every day*). Emotional exhaustion comprised items 1, 2, 3, 6, 8, 13, 14, 16, and 20 (range 0–54); depersonalization comprised items 5, 10, 11, 15, and 22 (range 0–30); and personal accomplishment comprised items 4, 7, 9, 12, 17, 18, 19, and 21 (range 0–48). Higher emotional‐exhaustion and depersonalization scores indicate greater burnout, whereas lower personal‐accomplishment scores indicate a less favorable state. No total MBI‐HSS composite was used. Cronbach’s alpha values were 0.947, 0.893, and 0.940, respectively.

#### 2.2.3. Fatigue

Fatigue was measured with the 14‐item Chalder Fatigue Scale [31, 53]. Items were binary coded 0/1; items 10, 13, and 14 were reverse scored as 1 minus the response. Scores range from 0 to 14, with higher scores indicating greater fatigue. Cronbach’s alpha was 0.824.

#### 2.2.4. Presenteeism‐Related Productivity Loss

Presenteeism‐related productivity loss was assessed with the Chinese Stanford Presenteeism Scale (SPS‐6) [40, 41, 43, 54, 55]. Six items are rated from 1 to 5. The analytic extract stored items 5 and 6 after reverse scoring, so all six stored scored items were summed directly. Scores range from 6 to 30, with higher values indicating greater productivity loss. Cronbach’s alpha was 0.919. Scoring and reliability of the focal measures are summarized in Supporting Table S3; their analytic roles and operational definitions are provided in Supporting Table S10.

#### 2.2.5. Covariates

The primary covariate set comprised sex, age, total nursing work duration, monthly income, education, and BMI. These variables were selected a priori to represent demographic, career‐exposure, socioeconomic, and health‐related characteristics that could be associated with both work–family balance and work functioning. Income and education were modeled as categorical factors rather than integer scores. Sensitivity analyses added marital status, monthly night‐shift frequency, the number of weeks exceeding 40 work hours in the prior month, employment type, professional title, management position, and survey year. A women‐only complete‐case sensitivity model also included previous birth; 761 of 3357 women had no usable parity value, so parity was not included in the primary model.

### 2.3. Statistical Analysis

Continuous variables are summarized with means and standard deviations and categorical variables with counts and percentages. Pearson correlations summarized linear relations among work–family balance, the three MBI‐HSS dimensions, fatigue, and productivity loss. With *N* = 3,597, correlations were interpreted descriptively rather than as tests of distributional normality.

Hierarchical linear regression modeled SPS‐6 productivity‐loss scores. Model 1 included work–family balance and the primary covariates; Model 2 added emotional exhaustion, depersonalization, and personal accomplishment simultaneously; and Model 3 added fatigue. The table reports unstandardized B coefficients, HC3 heteroscedasticity‐robust standard errors and 95% confidence intervals (CIs), and standardized beta coefficients for the core continuous variables. A joint HC3 Wald test evaluated the three MBI‐HSS dimensions, and Benjamini–Hochberg q values accompanied dimension‐specific tests. Categorical covariates were factor coded. Adjusted generalized variance‐inflation factors assessed collinearity.

The generalized model estimated work–family balance associations with each MBI‐HSS dimension, fatigue conditional on the three dimensions, and productivity loss conditional on work–family balance, the three dimensions, and fatigue. The total statistical indirect association was decomposed into seven nonoverlapping components: three dimension‐only paths, fatigue alone, and three serial paths through a dimension followed by fatigue. Individual‐level nonparametric bootstrap resampling with 5000 repetitions generated bias‐corrected percentile 95% CIs. Percentages were descriptive ratios to the total association. The analysis follows regression‐based multiple‐mediator methods [48, 49] and AGReMA reporting principles [56]; an interval excluding zero does not prove temporal mediation.

Sensitivity analyses used an extended covariate set, a women‐only parity‐adjusted model, survey‐year interaction models, regression diagnostics, and illustrative residual‐correlation analyses for each single‐dimension indirect component. Hospital identifiers and hospital‐level characteristics were unavailable; hospital‐clustered standard errors and multilevel models therefore could not be estimated. HC3 inference addresses heteroscedasticity, not within‐hospital dependence. Analyses were performed using R 4.5.0. Tests were two‐sided, with *p* < 0.05; dimension‐specific *q* values used the Benjamini–Hochberg procedure.

## 3. Results

### 3.1. Sample Flow and Participant Characteristics

The present study included 3597 nurses (Supporting Table S1). The mean age was 33.67 years (SD 6.83), mean nursing work duration was 10.59 years (SD 7.14), and mean BMI was 23.90 kg/m^2^ (SD 6.06). Most participants were women (93.3%), married (61.3%), and on a rotating day/night schedule (80.5%). Data were collected from 2021 through 2025; 2023 contributed the largest proportion (39.3%). Among 2596 women with usable parity data, 55.5% reported at least one previous birth (Table 1). Characteristics of included and quality‐control‐excluded emergency records are compared in Supporting Table S2.

**TABLE 1:** Overall demographic and occupational characteristics (*N* = 3597).

Characteristic	Category	Mean (SD) or *n* (%)
Age	Mean (SD), years	33.67 (6.83)
Total nursing work duration	Mean (SD), years	10.59 (7.14)
Body mass index	Mean (SD), kg/m^2^	23.90 (6.06)
Sex	Male	240 (6.7%)
Sex	Female	3357 (93.3%)
Marital status	Married	2205 (61.3%)
Marital status	Other	1392 (38.7%)
Education	Secondary technical	583 (16.2%)
Education	Junior college	1893 (52.6%)
Education	Bachelor	1109 (30.8%)
Education	Master or higher	12 (0.3%)
Rotating day/night schedule in prior 6 months	No	703 (19.5%)
Rotating day/night schedule in prior 6 months	Yes	2894 (80.5%)
Average night‐shift frequency	Day‐only/not applicable	676 (18.8%)
Average night‐shift frequency	≤ 4 per month	245 (6.8%)
Average night‐shift frequency	5–9 per month	1723 (47.9%)
Average night‐shift frequency	≥ 10 per month	953 (26.5%)
Weeks exceeding 40 work hours in prior month	0 week	896 (24.9%)
Weeks exceeding 40 work hours in prior month	1 week	622 (17.3%)
Weeks exceeding 40 work hours in prior month	2 weeks	586 (16.3%)
Weeks exceeding 40 work hours in prior month	3 weeks	305 (8.5%)
Weeks exceeding 40 work hours in prior month	4 weeks	1188 (33.0%)
Employment type	Permanent	577 (16.0%)
Employment type	Direct contract	2808 (78.1%)
Employment type	Agency	212 (5.9%)
Professional title	Junior	2289 (63.6%)
Professional title	Intermediate	1167 (32.4%)
Professional title	Senior	141 (3.9%)
Monthly income, yuan	< 3000	250 (7.0%)
Monthly income, yuan	3000–9000	2692 (74.8%)
Monthly income, yuan	> 9000	655 (18.2%)
Position	Staff nurse	3351 (93.2%)
Position	Ward‐level manager	231 (6.4%)
Position	Senior management	15 (0.4%)
Survey year	2021	473 (13.1%)
Survey year	2022	608 (16.9%)
Survey year	2023	1412 (39.3%)
Survey year	2024	638 (17.7%)
Survey year	2025	466 (13.0%)
Previous birth among women	No previous birth	1155 (44.5%)
Previous birth among women	At least one previous birth	1441 (55.5%)

*Note:* Values are mean (SD) or *n* (%). Percentages use nonmissing denominators. Previous birth is reported among women with usable parity data (*n* = 2596; missing *n* = 761).

Abbreviation: BMI, body mass index.

### 3.2. Scale Distributions and Correlations

Mean scores were 45.09 (SD 7.99) for work–family balance, 13.01 (SD 13.18) for emotional exhaustion, 4.93 (SD 6.47) for depersonalization, 21.42 (SD 15.59) for personal accomplishment, 7.87 (SD 3.68) for fatigue, and 14.83 (SD 6.16) for productivity loss. Work–family balance correlated with emotional exhaustion (*r* = −0.437), depersonalization (*r* = −0.366), personal accomplishment (*r* = 0.136), fatigue (*r* = −0.465), and productivity loss (*r* = −0.399). Emotional exhaustion and depersonalization were strongly correlated (*r* = 0.813) (Table 2).

**TABLE 2:** Descriptive statistics, reliability, and Pearson correlations (*N* = 3597).

Variable	Range	Mean (SD)	Alpha	1	2	3	4	5
Work–family balance	14–70; 14–70	45.09 (7.99)	0.745					
Emotional exhaustion	0–54; 0–54	13.01 (13.18)	0.947	−0.437				
Depersonalization	0–30; 0–30	4.93 (6.47)	0.893	−0.366	0.813			
Personal accomplishment	0–48; 0–48	21.42 (15.59)	0.940	0.136	0.395	0.292		
Fatigue	0–14; 0–14	7.87 (3.68)	0.824	−0.465	0.440	0.336	−0.011	
Presenteeism‐related productivity loss	6–30; 6–30	14.83 (6.16)	0.919	−0.399	0.472	0.398	0.156	0.378

*Note:* Values below the diagonal are Pearson correlations. All displayed nonzero correlations except personal accomplishment with fatigue (*r* = −0.011) had *p* < 0.001. Alpha, Cronbach’s alpha. Range gives possible and observed values. Higher SPS‐6 values denote greater productivity loss.

### 3.3. Hierarchical Regression

In Model 1, work–family balance was inversely associated with productivity loss (*B* = −0.307, HC3 95% CI ‐0.329–−0.285; beta = −0.398). Adding the three MBI‐HSS dimensions increased adjusted *R* ^2^ from 0.165 to 0.275 (delta *R* ^2^ = 0.110); the dimensions were jointly associated with productivity loss (*F*(3, 3582) = 99.77, *p* < 0.001). In Model 3, work–family balance (*B* = −0.163), emotional exhaustion (*B* = 0.113), depersonalization (*B* = 0.049), personal accomplishment (*B* = 0.028), and fatigue (*B* = 0.254) were associated with productivity loss. Adjusted *R* ^2^ was 0.290 (Table 3). Full primary Model 3 coefficients, including covariates, are reported in Supporting Table S4.

**TABLE 3:** Hierarchical regression with separate MBI‐HSS dimensions (*N* = 3597).

Model	Predictor	*B*	HC3 SE	95% CI	Beta	*p*	*q*
Model 1	Intercept	25.810	1.239	23.382 to 28.238		< 0.001	
Model 1	Work–family balance	−0.307	0.011	−0.329 to −0.285	−0.398	< 0.001	
Model 2	Intercept	18.942	1.237	16.517 to 21.366		< 0.001	
Model 2	Work–family balance	−0.197	0.013	−0.223 to −0.171	−0.256	< 0.001	
Model 2	Emotional exhaustion	0.144	0.013	0.118 to 0.170	0.308	< 0.001	< 0.001
Model 2	Depersonalization	0.037	0.024	−0.011 to 0.085	0.039	0.126	0.126
Model 2	Personal accomplishment	0.020	0.007	0.007 to 0.032	0.050	0.003	0.004
Model 3	Intercept	16.348	1.267	13.864 to 18.831		< 0.001	
Model 3	Work–family balance	−0.163	0.014	−0.190 to −0.137	−0.212	< 0.001	
Model 3	Emotional exhaustion	0.113	0.014	0.087 to 0.140	0.243	< 0.001	< 0.001
Model 3	Depersonalization	0.049	0.024	0.001 to 0.096	0.051	0.044	0.044
Model 3	Personal accomplishment	0.028	0.007	0.015 to 0.040	0.070	< 0.001	< 0.001
Model 3	Fatigue	0.254	0.029	0.196 to 0.311	0.152	< 0.001	

*Note:* All models adjusted for sex, age, total nursing work duration, income, education, and BMI. Income and education were factor coded. *B* is unstandardized; beta is standardized for displayed continuous variables. Model 1 *R* ^2^ = 0.167; Model 2 *R* ^2^ = 0.277 (delta *R* ^2^ = 0.110); and Model 3 *R* ^2^ = 0.293 (delta *R* ^2^ = 0.016). *q* values are Benjamini–Hochberg adjusted within each model for the three MBI‐HSS dimensions.

### 3.4. Statistical Indirect Associations

The adjusted total association of work–family balance with productivity loss was −0.3069, and the direct association conditional on the three MBI‐HSS dimensions and fatigue was −0.1634. The total statistical indirect association was −0.1435 (95% bias‐corrected bootstrap CI −0.1621–−0.1257), or 46.8% of the total association. Dimension‐only components summed to −0.0890 (29.0%): emotional exhaustion −0.0819, depersonalization −0.0144, and personal accomplishment +0.0073. Fatigue alone contributed −0.0338 (11.0%). The three dimension‐to‐fatigue serial components summed to −0.0206 (6.7%) (Table 4 and Figure 1). Individual path coefficients with HC3 inference are reported in Supporting Table S8.

**TABLE 4:** Adjusted direct and statistical indirect associations (*N* = 3597).

Effect	*B*	Boot SE	BC 95% CI	Std effect	% total	*q*
Total association	−0.3069	0.0110	−0.3284 to −0.2848	−0.3980		
Direct association conditional on the three MBI‐HSS domains and fatigue	−0.1634	0.0137	−0.1906 to −0.1371	−0.2119		
Total statistical indirect association	−0.1435	0.0093	−0.1621 to −0.1257	−0.1860	46.8%	
Combined domain‐only components	−0.0890	0.0088	−0.1066 to −0.0718	−0.1154	29.0%	
Via emotional exhaustion alone	−0.0819	0.0103	−0.1017 to −0.0621	−0.1062	26.7%	< 0.001
Via depersonalization alone	−0.0144	0.0071	−0.0284 to −0.0006	−0.0187	4.7%	0.040
Via personal accomplishment alone	0.0073	0.0020	0.0037 to 0.0116	0.0094	−2.4%	< 0.001
Via fatigue alone	−0.0338	0.0045	−0.0434 to −0.0256	−0.0439	11.0%	< 0.001
Combined serial components through an MBI‐HSS domain then fatigue	−0.0206	0.0027	−0.0265 to −0.0160	−0.0268	6.7%	
Via emotional exhaustion then fatigue	−0.0219	0.0029	−0.0281 to −0.0168	−0.0284	7.1%	< 0.001
Via depersonalization then fatigue	0.0034	0.0011	0.0015 to 0.0058	0.0044	−1.1%	< 0.001
Via personal accomplishment then fatigue	−0.0021	0.0005	−0.0031 to −0.0014	−0.0027	0.7%	< 0.001

*Note:* All components adjusted for sex, age, total nursing work duration, income, education, and BMI. BC, bias corrected; 5000 individual‐level nonparametric bootstrap repetitions. Percentages are descriptive ratios to the total association and are not causal proportions mediated. Dimension‐specific *q* values use the Benjamini–Hochberg method. The positive personal‐accomplishment‐only and depersonalization‐then‐fatigue components are countervailing components.

**FIGURE 1:**
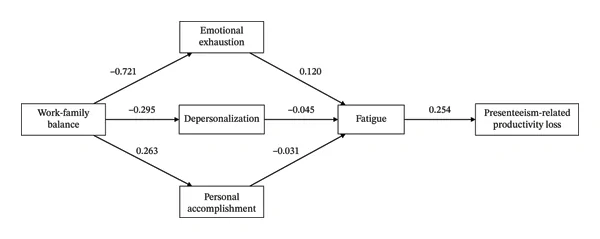
Simplified cross‐sectional indirect‐association diagram. Values on displayed paths are unstandardized coefficients adjusted for sex, age, total nursing work duration, income, education, and BMI. For visual clarity, the following paths are not drawn: work–family balance to fatigue conditional on the three MBI‐HSS dimensions (*B* = −0.133), each dimension to productivity loss, and work–family balance to productivity loss conditional on the dimensions and fatigue. Complete estimates are reported in Tables 3 and 4 and Supporting Table S8.

### 3.5. Diagnostics and Sensitivity Analyses

Adjusted generalized variance‐inflation factors ranged from 1.006 to 2.552 (Supporting Table S5). The largest values were for age and nursing work duration; the three MBI‐HSS dimensions ranged from 1.198 to 1.999. The Breusch–Pagan test indicated heteroscedasticity (*p* < 0.001), supporting HC3 inference. Maximum Cook’s distance was 0.010; diagnostic plots are shown in Supporting Figure S1.

In the extended all‐participant model, coefficients were similar for work–family balance (*B* = −0.154), emotional exhaustion (*B* = 0.110), depersonalization (*B* = 0.047), personal accomplishment (*B* = 0.029), and fatigue (*B* = 0.242). In 2596 women with complete parity data, corresponding estimates were −0.147, 0.098, 0.069, 0.034, and 0.266. The depersonalization estimate in the extended model was attenuated (*p* = 0.052); the other core estimates remained statistically compatible with nonzero associations (Supporting Table S6).

The work–family balance coefficient was inverse in every survey year (*B* range −0.329–−0.247), and the omnibus interaction with year was not statistically significant (*F*(4, 3564) = 1.43, *p* = 0.222) (Supporting Table S7).

Illustrative single‐dimension sensitivity analyses indicated that the estimated indirect association reached zero at residual mediator‐outcome correlations of approximately 0.36 for emotional exhaustion, 0.30 for depersonalization, and +0.22 for personal accomplishment (Supporting Table S9). These analyses do not identify the full generalized decomposition or address all forms of unmeasured confounding.

## 4. Discussion

In this large convenience sample of emergency nurses, better work–family balance was associated with lower presenteeism‐related productivity loss. The three MBI‐HSS dimensions added substantial explanatory information as a set, and emotional exhaustion accounted for the largest dimension‐specific indirect component. Fatigue contributed independently, while the combined serial component was smaller. These are cross‐sectional statistical components, not evidence of a temporal mechanism.

Work–family balance may reflect access to recovery, family support, and perceived control, which COR theory treats as valued resources [9, 10]. JD–R theory places these resources within a work system containing demands such as high acuity, emotional labor, staffing pressure, and irregular schedules [11, 12]. Studies linking work–family conflict with burnout in nurses support examining these relations [16–20], but the present data cannot determine whether work conditions, family conditions, or personal health preceded the measured scores.

Emotional exhaustion showed the largest dimension‐only component. This accords with evidence that exhaustion is closely related to occupational distress and work functioning in emergency nurses [21–30]. Depersonalization contributed a smaller component, and its estimate was sensitive to the extended covariate set. The high correlation between emotional exhaustion and depersonalization also means that their mutually adjusted coefficients should be interpreted as dimension‐specific conditional associations, not independent causal effects.

Personal accomplishment showed a small countervailing dimension‐only component, whereas its serial component through fatigue was inverse. The positive conditional outcome coefficient should not be interpreted as greater accomplishment causing productivity loss; it may reflect suppression among correlated MBI‐HSS dimensions or unmeasured differences in work role and response patterns. Reporting this result separately is preferable to concealing it within a total burnout composite.

Fatigue contributed an inverse fatigue‐only component and a smaller combined serial component. The model placed the MBI‐HSS dimensions before fatigue to examine a resource‐depletion hypothesis, but fatigue‐first, parallel, and reciprocal structures remain plausible. Repeated measurements within nurses and shifts are needed to distinguish accumulation, recovery, and reciprocal change.

For nursing management, the findings identify organizational targets for prospective evaluation. Predictable rosters, transparent night‐shift allocation, adequate staffing, protected recovery time, and family‐supportive supervision address demands and resources described by COR and JD–R theory. Their effects on burnout dimensions, fatigue, and productivity loss should be tested in cluster‐aware prospective studies. Recommendations should not depend on the smaller serial component alone.

Associations remained inverse in every survey year, but their magnitude varied. The 2021–2025 period spans changing COVID‐19 pressures, workforce policies, and service conditions. Survey year adjustment cannot capture those contextual changes; repeated cohort observations and hospital‐level data would be more informative.

### 4.1. Strengths and Limitations

Strengths include a large emergency‐nurse sample, explicit reconstruction of the analytic flow, separate scoring of the three MBI‐HSS dimensions, factor coding of categorical covariates, HC3 inference, 5000 bootstrap repetitions, and prespecified sensitivity analyses.

The study has important limitations. First, the cross‐sectional design cannot establish exposure‐mediator‐outcome timing, mediation, or causation; reverse and reciprocal relations remain possible. Second, all focal measures were self‐reported at the same occasion, creating risks of recall error and common‐method variance. Third, hospitals and participants were recruited by convenience, invitation denominators were unavailable, and nonresponse and healthy‐worker bias could not be quantified. Fourth, hospital identifiers and hospital‐level characteristics were unavailable, so intracluster correlation, multilevel models, and hospital‐clustered standard errors could not be estimated. Fifth, residual confounding is likely because staffing ratios, workload intensity, sleep, family support, workplace violence, chronic disease, and other contextual variables were unavailable or incomplete. Sixth, the 2021–2025 period spans changing pandemic and postpandemic conditions that survey year cannot fully capture. Seventh, the strong correlation between emotional exhaustion and depersonalization and the countervailing personal‐accomplishment component indicate that dimension‐specific conditional estimates may include suppression and require replication.

## 5. Conclusions

Among 3597 emergency nurses, better work–family balance was associated with lower presenteeism‐related productivity loss. Emotional exhaustion accounted for the largest dimension‐specific indirect component, fatigue contributed independently, and the combined serial component was smaller. The separate personal‐accomplishment result showed why a total MBI‐HSS composite would be difficult to interpret. Prospective studies with repeated measurements and hospital clustering are needed before temporal or practice effects are inferred.

## Author Contributions

Fangyun Luan: conceptualization, methodology, investigation, data curation, formal analysis, and writing–original draft. Jingzheng Yan: methodology, formal analysis, validation, and writing–review and editing. Wenlong Sun: investigation, data collection, visualization, and writing–review and editing. Xiao Han: investigation, resources, validation, and writing–review and editing. Yingjuan Cao: conceptualization, supervision, project administration, resources, and writing–review and editing.

## Funding

No dedicated funding supported this work.

## Disclosure

All authors have read and approved the final manuscript.

## Ethics Statement

The Shandong Nurses’ Health Cohort was approved by the Research Ethics Committee of Qilu Hospital of Shandong University (approval No. KYLL‐202011‐085). Participant recruitment and data collection began after this approval document was issued. The cohort was subsequently expanded in sample size and geographical recruitment scope to establish the Chinese Nurses’ Health Cohort (approval No. KYLL‐202210‐060‐4). All participants provided electronic informed consent.

## Consent

Please see the Ethics Statement.

## Conflicts of Interest

The authors declare no conflicts of interest.

## Supporting Information

Additional supporting information can be found online in the Supporting Information section.

## Supporting information


**Supporting Information** Supporting Tables S1 and S2 document the reconstructed analytic flow and the comparison of included and quality‐control‐excluded emergency records. Supporting Tables S3 and S10 describe measure scoring, reliability, and variable operationalization. Supporting Tables S4–S9 provide full regression and path coefficients, multicollinearity diagnostics, and sensitivity analyses. Supporting Figure S1 presents diagnostic plots for primary Model 3.

## Data Availability

Data are not publicly posted because institutional access requirements apply. Requests may be directed to the corresponding author and will be considered under those requirements.
