# Supplementary material for: Work–Family Balance and Presenteeism‐Related Productivity Loss in Emergency Nurses: Cross‐Sectional Indirect Associations Through Burnout Dimensions and Fatigue
Source: J Nurs Manag. 2026 Oct 26;2026:7344295. doi: 10.1155/jonm/7344295 (PMC13710889; doi:10.1155/jonm/7344295)
Supplement: Supplementary file 1 [file JONM-2026-7344295-s001.docx]

**Supplementary Material**

Work-Family Balance and Presenteeism-Related Productivity Loss in Emergency Nurses: Cross-Sectional Indirect Associations Through Burnout Dimensions and Fatigue

# **Supplementary Table S1. Reconstructed analytic flow**

| **Stage** | **n** |
| --- | --- |
| Records in de-identified cohort master | 64114 |
| Emergency-department records | 3920 |
| Excluded: BMI missing | 14 |
| Emergency-department records with BMI available | 3906 |
| Excluded by pre-existing cohort validity/quality-control processing | 309 |
| Final analytic sample | 3597 |

*Quality-control criteria could overlap, and participant-level rule indicators were unavailable.*

# **Supplementary Table S2. Comparison of included and quality-control-excluded emergency records**

| **Characteristic** | **Category** | **Group** | **n (%)** | **Overall P** |
| --- | --- | --- | --- | --- |
| Sex | Male | Excluded after quality control | 20 (6.5%) | .987 |
| Sex | Male | Included | 240 (6.7%) | .987 |
| Sex | Female | Excluded after quality control | 289 (93.5%) | .987 |
| Sex | Female | Included | 3,357 (93.3%) | .987 |
| Marital status | Married | Excluded after quality control | 166 (53.7%) | .011 |
| Marital status | Married | Included | 2,205 (61.3%) | .011 |
| Marital status | Other | Excluded after quality control | 143 (46.3%) | .011 |
| Marital status | Other | Included | 1,392 (38.7%) | .011 |
| Rotating day/night schedule | No | Excluded after quality control | 64 (20.7%) | .674 |
| Rotating day/night schedule | No | Included | 703 (19.5%) | .674 |
| Rotating day/night schedule | Yes | Excluded after quality control | 245 (79.3%) | .674 |
| Rotating day/night schedule | Yes | Included | 2,894 (80.5%) | .674 |
| Survey year | 2021 | Excluded after quality control | 35 (11.3%) | .172 |
| Survey year | 2021 | Included | 473 (13.1%) | .172 |
| Survey year | 2022 | Excluded after quality control | 62 (20.1%) | .172 |
| Survey year | 2022 | Included | 608 (16.9%) | .172 |
| Survey year | 2023 | Excluded after quality control | 109 (35.3%) | .172 |
| Survey year | 2023 | Included | 1,412 (39.3%) | .172 |
| Survey year | 2024 | Excluded after quality control | 52 (16.8%) | .172 |
| Survey year | 2024 | Included | 638 (17.7%) | .172 |
| Survey year | 2025 | Excluded after quality control | 51 (16.5%) | .172 |
| Survey year | 2025 | Included | 466 (13.0%) | .172 |

*Sex coding was verified as male/female. Marital categories other than married were combined as Other. The unverified raw educational-qualification code was omitted rather than assigned speculative labels.*

# **Supplementary Table S3. Reliability and scoring of focal measures**

| **Scale/domain** | **Items** | **Range** | **Cronbach alpha** | **Direction** |
| --- | --- | --- | --- | --- |
| Work-family balance | 14 | 14-70 | 0.745 | Higher = more favorable balance |
| Emotional exhaustion | 9 | 0-54 | 0.947 | Higher = greater exhaustion |
| Depersonalization | 5 | 0-30 | 0.893 | Higher = greater depersonalization |
| Personal accomplishment | 8 | 0-48 | 0.940 | Lower = less favorable accomplishment |
| Fatigue | 14 | 0-14 | 0.824 | Higher = greater fatigue |
| SPS-6 productivity loss | 6 | 6-30 | 0.919 | Higher = greater productivity loss |

# **Supplementary Table S4. Full primary Model 3 coefficients with HC3 inference**

| **Term** | **B** | **HC3 SE** | **95% CI** | **P** |
| --- | --- | --- | --- | --- |
| Intercept | 16.348 | 1.267 | 13.864 to 18.831 | <.001 |
| Work-family balance | -0.163 | 0.014 | -0.190 to -0.137 | <.001 |
| Emotional exhaustion | 0.113 | 0.014 | 0.087 to 0.140 | <.001 |
| Depersonalization | 0.049 | 0.024 | 0.001 to 0.096 | .044 |
| Personal accomplishment | 0.028 | 0.007 | 0.015 to 0.040 | <.001 |
| Fatigue | 0.254 | 0.029 | 0.196 to 0.311 | <.001 |
| Sex: female (vs male) | 0.139 | 0.326 | -0.501 to 0.778 | .671 |
| Age, years | -0.011 | 0.030 | -0.070 to 0.048 | .720 |
| Nursing work duration, years | 0.064 | 0.029 | 0.007 to 0.120 | .027 |
| Income: 3,000-9,000 (vs <3,000 yuan) | 0.059 | 0.381 | -0.687 to 0.806 | .876 |
| Income: >9,000 (vs <3,000 yuan) | -0.038 | 0.437 | -0.895 to 0.818 | .930 |
| Education: junior college (vs secondary technical) | 0.449 | 0.284 | -0.107 to 1.004 | .113 |
| Education: bachelor (vs secondary technical) | 0.463 | 0.322 | -0.167 to 1.094 | .150 |
| Education: master or higher (vs secondary technical) | 0.209 | 1.402 | -2.538 to 2.956 | .881 |
| BMI, kg/m² | 0.029 | 0.016 | -0.002 to 0.059 | .066 |

# **Supplementary Table S5. Multicollinearity diagnostics**

| **Term** | **GVIF** | **df** | **Adjusted GVIF** |
| --- | --- | --- | --- |
| Work-family balance | 1.565 | 1 | 1.251 |
| Emotional exhaustion | 3.995 | 1 | 1.999 |
| Depersonalization | 2.998 | 1 | 1.731 |
| Personal accomplishment | 1.435 | 1 | 1.198 |
| Fatigue | 1.453 | 1 | 1.205 |
| gender | 1.012 | 1 | 1.006 |
| Age, years | 6.331 | 1 | 2.516 |
| Nursing work duration, years | 6.511 | 1 | 2.552 |
| income3 | 1.151 | 2 | 1.036 |
| education | 1.560 | 3 | 1.077 |
| BMI, kg/m² | 1.028 | 1 | 1.014 |

*Adjusted GVIF is GVIF^(1/[2 x df]). Maximum adjusted GVIF=2.552.*

# **Supplementary Table S6. Extended-covariate and parity sensitivity models**

| **Model** | **Term** | **n** | **B** | **HC3 SE** | **95% CI** | **beta** | **P** | **q** |
| --- | --- | --- | --- | --- | --- | --- | --- | --- |
| Extended all-participant model | Work-family balance | 3597 | -0.154 | 0.014 | -0.181 to -0.127 | -0.200 | <.001 |  |
| Extended all-participant model | Emotional exhaustion | 3597 | 0.110 | 0.014 | 0.084 to 0.137 | 0.236 | <.001 | <.001 |
| Extended all-participant model | Depersonalization | 3597 | 0.047 | 0.024 | -0.000 to 0.094 | 0.049 | .052 | .052 |
| Extended all-participant model | Personal accomplishment | 3597 | 0.029 | 0.006 | 0.016 to 0.042 | 0.073 | <.001 | <.001 |
| Extended all-participant model | Fatigue | 3597 | 0.242 | 0.029 | 0.184 to 0.300 | 0.145 | <.001 |  |
| Female complete-parity sensitivity model | Work-family balance | 2596 | -0.147 | 0.016 | -0.179 to -0.115 | -0.191 | <.001 |  |
| Female complete-parity sensitivity model | Emotional exhaustion | 2596 | 0.098 | 0.016 | 0.067 to 0.129 | 0.207 | <.001 | <.001 |
| Female complete-parity sensitivity model | Depersonalization | 2596 | 0.069 | 0.028 | 0.014 to 0.124 | 0.070 | .014 | .014 |
| Female complete-parity sensitivity model | Personal accomplishment | 2596 | 0.034 | 0.008 | 0.019 to 0.049 | 0.086 | <.001 | <.001 |
| Female complete-parity sensitivity model | Fatigue | 2596 | 0.266 | 0.035 | 0.199 to 0.334 | 0.160 | <.001 |  |

# **Supplementary Table S7. Survey-year sensitivity of the work-family balance coefficient**

| **Survey year** | **B** | **HC3 SE** | **95% CI** | **P** |
| --- | --- | --- | --- | --- |
| 2021 | -0.282 | 0.030 | -0.341 to -0.223 | <.001 |
| 2022 | -0.329 | 0.030 | -0.388 to -0.270 | <.001 |
| 2023 | -0.289 | 0.019 | -0.325 to -0.252 | <.001 |
| 2024 | -0.247 | 0.025 | -0.296 to -0.198 | <.001 |
| 2025 | -0.250 | 0.030 | -0.308 to -0.191 | <.001 |

*Omnibus work-family balance x survey-year interaction: F(4,3564)=1.427, P=.222.*

# **Supplementary Table S8. HC3 path coefficients in the generalized model**

| **Path** | **B** | **HC3 SE** | **95% CI** | **P** |
| --- | --- | --- | --- | --- |
| WFB to emotional exhaustion | -0.721 | 0.024 | -0.768 to -0.675 | <.001 |
| WFB to depersonalization | -0.295 | 0.013 | -0.320 to -0.271 | <.001 |
| WFB to personal accomplishment | 0.263 | 0.035 | 0.195 to 0.331 | <.001 |
| WFB to fatigue conditional on MBI-HSS domains | -0.133 | 0.008 | -0.149 to -0.118 | <.001 |
| Emotional exhaustion to fatigue | 0.120 | 0.007 | 0.106 to 0.133 | <.001 |
| Depersonalization to fatigue | -0.045 | 0.013 | -0.070 to -0.019 | <.001 |
| Personal accomplishment to fatigue | -0.031 | 0.004 | -0.039 to -0.024 | <.001 |
| Emotional exhaustion to productivity loss | 0.113 | 0.014 | 0.087 to 0.140 | <.001 |
| Depersonalization to productivity loss | 0.049 | 0.024 | 0.001 to 0.096 | .044 |
| Personal accomplishment to productivity loss | 0.028 | 0.007 | 0.015 to 0.040 | <.001 |
| Fatigue to productivity loss | 0.254 | 0.029 | 0.196 to 0.311 | <.001 |
| Direct WFB-productivity-loss association | -0.163 | 0.014 | -0.190 to -0.137 | <.001 |
| Total WFB-productivity-loss association | -0.307 | 0.011 | -0.329 to -0.285 | <.001 |

# **Supplementary Table S9. Illustrative residual-correlation sensitivity analyses**

| **Single-domain illustration** | **rho at zero** | **R²* product** | **R²~ product** |
| --- | --- | --- | --- |
| Emotional exhaustion | 0.36 | 0.1296 | 0.0751 |
| Depersonalization | 0.30 | 0.0900 | 0.0587 |
| Personal accomplishment | 0.22 | 0.0484 | 0.0367 |

*These single-domain illustrations do not identify the full generalized decomposition and do not address all unmeasured confounding.*

# **Supplementary Table S10. Operationalization of analysis variables**

| **Variable** | **Analytic role** | **Operationalization** | **Direction** |
| --- | --- | --- | --- |
| Work-family balance | Exposure | 14-item total after reversing interference items | 14-70; higher = more favorable |
| Emotional exhaustion | MBI-HSS dimension | Sum of 9 standard items | 0-54; higher = greater exhaustion |
| Depersonalization | MBI-HSS dimension | Sum of 5 standard items | 0-30; higher = greater depersonalization |
| Personal accomplishment | MBI-HSS dimension | Sum of 8 standard items; no total burnout composite | 0-48; lower = less favorable |
| Fatigue | Downstream correlate | 14 binary items after specified reverse coding | 0-14; higher = greater fatigue |
| SPS-6 productivity loss | Outcome | Sum of 6 stored scored items | 6-30; higher = greater loss |

# **Supplementary Figure S1. Primary MBI-domain regression diagnostics**

*Plots are based on primary hierarchical regression Model 3.*
